# A fast read alignment method based on seed-and-vote for next generation sequencing

**DOI:** 10.1186/s12859-016-1329-6

**Published:** 2016-12-23

**Authors:** Song Liu, Yi Wang, Fei Wang

**Affiliations:** 1 Shanghai Key Lab of Intelligent Information Processing, Shanghai, China; 2 School of Computer Science and Technology, Fudan University, Shanghai, China; 3 School of Life Sciences, Fudan University, Shanghai, China

**Keywords:** Read alignment, Seed and vote, Hash table

## Abstract

**Background:**

The next-generation of sequencing technologies, along with the development of bioinformatics, are generating a growing number of reads every day. For the convenience of further research, these reads should be aligned to the reference genome by read alignment tools. Despite the diversity of read alignment tools, most have no comprehensive advantage in both accuracy and speed. For example, BWA has comparatively high accuracy, but its speed leaves much to be desired, becoming a bottleneck while an increasing number of reads need to be aligned every day. We believe that the speed of read alignment tools still has huge room for improvement, while maintaining little to no loss in accuracy.

**Results:**

Here we implement a new read alignment tool, Fast Seed-and-Vote Aligner (FSVA), which is based on seeding and voting. FSVA achieves a high accuracy close to BWA and simultaneously has a very high speed. It only requires ~10–15 CPU hours to run a whole genome read alignment, which is ~5–7 times faster than BWA.

**Conclusions:**

In some cases, reads have to be aligned in a short time. Where requirement of accuracy is not very stringent, FSVA would be a promising option.

FSVA is available at https://github.com/Topwood91/FSVA

## Background

Next-generation sequencing technologies have developed rapidly in recent years mainly in two regards. On the one hand, the throughput potential is tremendous. For example, a system consisting of a set of 10 HiSeq X ultra-high-throughput instruments (HiSeq X10) can deliver over 18,000 human genomes per year. On the other hand, the cost of whole genome sequencing is decreasing steadily. Currently the cost of whole genome sequencing for an individual or patient stands at roughly $1,000. It seems likely that this trend will continue, and sequencing costs will continue to fall. This allows access to genome sequencing for a large percentage of the population. All of these changes have led to a sharp increase in the amount of sequence data and pose a new challenge to sequence analyzers.

Usually, the data produced by a sequencing platform is not a single sequence with all DNA information, but consists instead of a large number of short subsequences, called reads, with partial DNA information. Read alignment is then required to map reads to a reference genome and identify the coordinate of each individual read on the reference. The past few years have witnessed the appearance of diverse read alignment tools, which can be roughly divided into two categories: tools based on hash table and tools based on prefix/suffix trie [1]. A tool from the first category usually builds a hash table for the genome reference, which enables a shorter part of the read (called seed) to be mapped to the genome in constant time. Then, the coordinate of the read is determined from the result of seed extension at each of its mapping locations. Representatives of this category are BLAST [2], SOAP [3] and MAQ [4]. A tool of the second category, on the other hand, usually searches the prefix/suffix trie of the genome and then calculates the coordinate of each individual read with the help of Burrows-Wheeler Transform [5]. Representatives of this category include BWA [6], Bowtie [7] and SOAP2 [8].

Read alignment is usually the first and most time consuming step of genome sequence analysis. Although some existing tools are widely used with great success, their speeds cannot keep up with data increases. The very widely-used BWA definitely has many advantages and achieves relatively accurate results, but its speed is not as fast as could be desired. Several new versions of BWA such as BWA-SW [9] and BWA-MEM [10] are still limited by low speeds. For example, using BWA-MEM to process a read alignment on a whole genome (the library size is ~200 GB) usually takes ~70–80 CPU hours when running on a single core. This means that 16–20 CPU cores are required to ensure the speed of read alignment can keep up with the speed of data generation for a HiSeq X10. Other tools have no prominent advantage in speed at the same accuracy level as BWA. Thus, speeding up the read alignment is of vital importance and can significantly improve the efficiency of sequence analysis.

To accelerate the speed, researchers have tried many methods, such as seeking assistance from GPU [11], cloud computing [12] and distributed computing [13]. But these methods usually have a high requirement for hardware and often cannot be implemented due to resource limitation. Naturally, improvement in algorithm is a better option, such as in Subread [14]. Subread is also based on hash table, which adopts a seed-and-vote strategy instead of the extending step of usual hash-table methods. Like many read alignment tools, Subread first builds an index for the reference genome, which enables a subread, a subsequence of a read, to identify its coordinate on the reference genome in constant time. Then, it extracts multiple subreads from each individual read, gets the coordinate of the subread on the reference genome, and uses the coordinates of the subread to vote the final mapping location of the read. Although the seed-and-vote strategy is time-saving, the mapping accuracy of Subread is rather unsatisfactory in practice. Besides, our tests on real data show that Subread does not work well with large sequence data (over 300GB). It produces one third of the output only, while running for more than 500 CPU hours.

In this study, we propose a new read alignment tool, Fast Seed-and-Vote Aligner (FSVA), which is ~5–7 times faster in running time than BWA-MEM while keeping a similar mapping accuracy as BWA-MEM. In practice, for a whole genome read alignment (library size ~200 GB), FSVA costs ~10–15 CPU hours on a single core and ~4–6 CPU hours on four cores. The respective time cost of BWA-MEM in the same scenario is about ~70 CPU hours and ~20 CPU hours. This advantage of speed makes FSVA a promising read alignment tool for big data.

FSVA, borrowing the seed-and-vote strategy, builds a hash table for a reference genome and extracts seeds from the read to vote the coordinate. Compared with Subread, the main improvement of FSVA lies in the longer seed, which allows improved running speed and accuracy. While a longer seed cannot be represented as an integer in many programming languages, we avoid this problem by expressing the seed as a large prime number, guaranteeing a seed can be represented as an integer, and the size of hash table is not too big. This specific method is introduced in detail in the METHODS section. Experiments on simulated data and real data illustrate the great advantages of FSVA on time saving and present the alignment accuracy of FSVA as close to that of BWA-MEM, which is shown in the RESULTS section.

## Methods

FSVA, based on a seed-and-vote strategy, extracts seeds from a read and makes them vote the coordinate of the read. This method includes two steps: indexing and voting. The detailed methods are described in following sections. One point to note is that in the METHODS section, our algorithm is introduced based on 150 bp reads, which is the read length of HiSeq X10. In the actual situation, our tool FSVA can automatically adjust its parameters to fit various read lengths.

### Building the index

Building the index refers to the building of a hash table for a reference genome sequence. In our hash table, the key is a 32bit unsigned integer converted from a subsequence of the reference genome. The value is a vector of 32bit unsigned integer, representing the location of the reference which a seed can be exactly mapped to.

#### Calculating keys

**Fig. 1:**
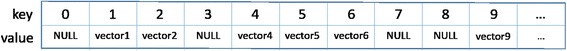
The process of key calculation. ${X}_i$ is a 62bit unsigned integer calculated by converting a 31 bp subsequence into a binary number. ${key}_i$ is a 32 bit unsigned integer, calculated by ${X}_i$ modulo a large prime number M

A DNA sequence, which usually contains only 4 characters (A, G, C, T), can be converted to a quaternary number. Thus an n-long DNA sequence can be converted to an unsigned quaternary integer with n digits, or an unsigned binary integer with 2n digits, namely, a 2n bit binary integer. Here we extract 31 bp subsequences from the beginning of a reference genome as keys, and the size of a sliding window of each pair of neighbor subsequences is set as 8 bp. To store a 31 bp subsequence, a 62bit unsigned binary integer is needed. For the sake of memory saving, we designate the 62bit unsigned integer modulo a large prime number M. Herein M is no bigger than the maximum of a 32bit unsigned integer. Thus the 62bit unsigned integer is converted to a 32bit one, which is the final key utilized in the hash table. If a 31 bp subsequence extracted from the reference genome consists of other characters (not A, C, G and T), it is dropped without key calculation. The process of key calculation is shown in Fig. 1.

#### Building the hash table

**Fig. 2:**
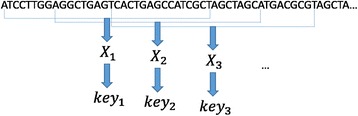
An example of our hash table. Vector*n* represents the vector of coordinates of subsequences with the same key *n*. If no subsequences with the key *m*, the value of *m* is NULL

The hash table has M pairs of key and value, with the key coming from the modulo operation. Since the key is calculated from converting an unsigned 62bit integer into a 32 bit one by modulo M operation, different subsequences may be given the same key. Therefore, a vector is utilized to store coordinates of the subsequences with the same key, and the value of the key is this vector. If no value exists for a key, we mark NULL. Figure 2 shows an example of the hash table.

### Generating seeds and voting

**Fig. 3:**
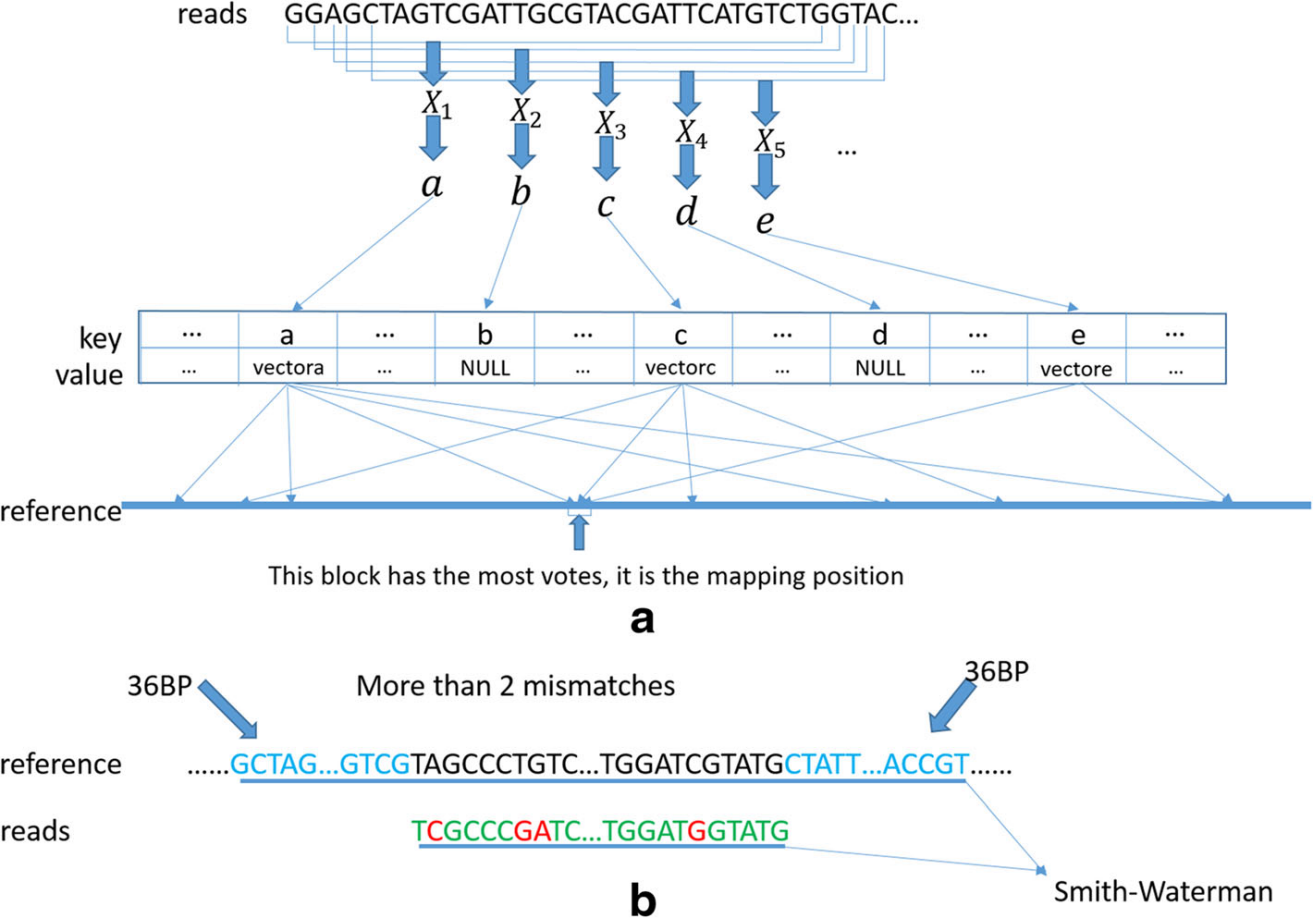
**a** The process of generating seeds and voting. a, b, c, d and e are determined by the same method of key calculation and used to query the hash table. If the value is not NULL, we can get a vector which stores some coordinates and then votes on each one of all coordinates in the value vector. After voting from all seeds is completed, the block with the most votes is selected as the mapping coordinate of the read. **b** In case of more than 2 mismatches between a read and its mapping block (block with the most votes), the mapping block is extended towards bi-directions with 36 bp, then Smith-Waterman algorithm is applied on the read and the extending block. In this figure, green, red, and blue represent a match, a mismatch, and the 36 bp upstream and downstream of the extending block, respectively

We treat each one 31 bp subsequence extracted from a read as a seed. Thus an n-long read can generate a total of n-30 different seeds. The process of key calculation of hash table building is utilized also to get the key of a seed. Then, by querying the hash table with the key of a seed, the location of the seed can be located. The seed set with size n-30 of an n-long read can search out its corresponding coordinate set with at most n-30 vectors. Coordinates recorded in these vectors vote the coordinate of the read, and the one with most votes is selected. Herein, the vote counting is based on a block. A block is an interval of the genome reference with same length of the read. Figure 3a shows the process of generating seeds and voting.

In practice, the final mapping block should have at least two votes; otherwise, we believe this read cannot be mapped to the reference genome. Second, if more than one block is tied for most votes, we choose one randomly and set its mapping quality as 0. Third, if the alignment has more than two mismatches, we perform a Smith-Waterman dynamic programming between the read and the extending block. The extending block is the block with the most votes extending towards upstream and downstream with 36 bp (Fig. 3b). Fourth, our experience from a range of experiments shows that 99% seeds will vote less than ~450 coordinates on the default condition (read length is 150 and seed length is 31), and if a seed votes more than 450 coordinates, we believe this seed is unrepresentative. These unrepresentative seeds are dropped to avoid time-wasting. This threshold of how many coordinates at most a seed can vote is a tradeoff between time cost and alignment accuracy. In our tool, this threshold can be set by users for specific requirements.

In our experiment, we set the length of seed as 31 bp, and for hash table also 31 bp subsequences are extracted from the genome reference to calculate keys. If seed is shorter, many seeds will be generated from a read and much more coordinates could be selected as candidates waiting to be voted upon. Consequently, the time cost is higher. On the other hand, if the seed length is larger than 32 bp, it cannot be converted to a 64bit integer and cannot be represented in most programming languages, which will introduce trouble on the programming side.

**Fig. 4:**
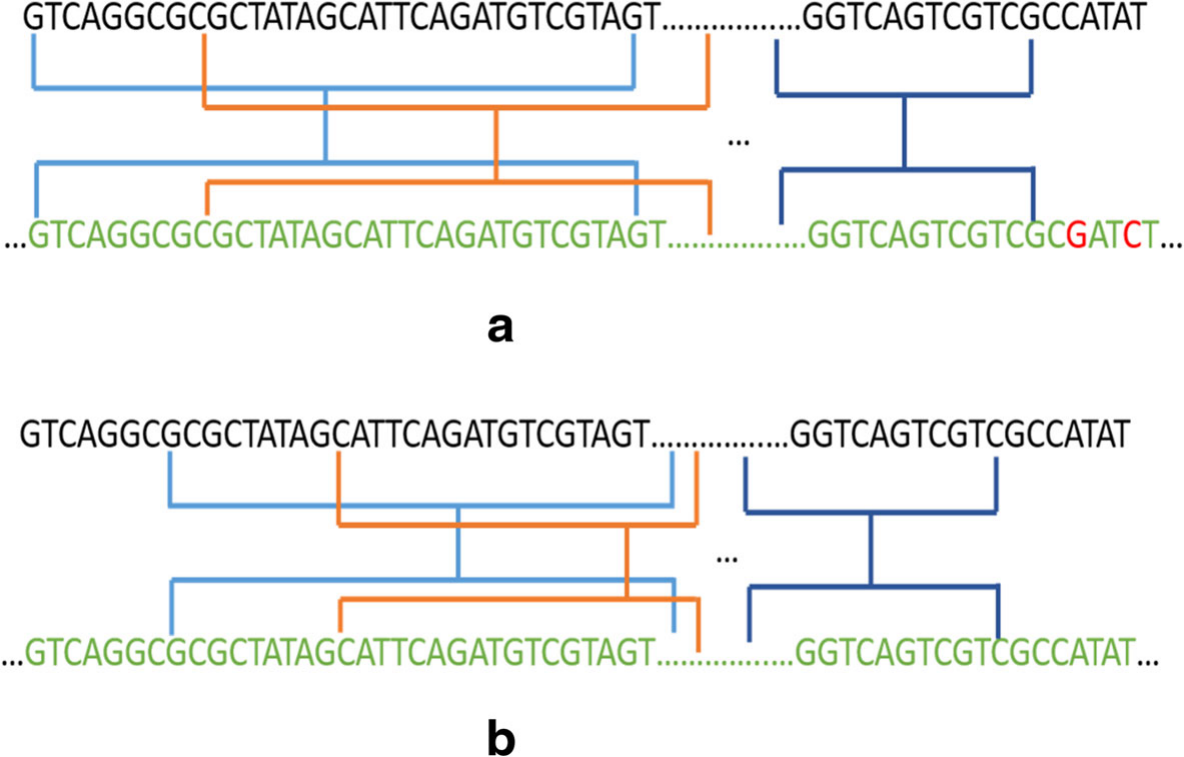
**a** A block having 2 mismatches. In this situation, the first seed of the read voting to the block starts at the first base pair. Since the gap between two neighbor seeds is 8, the seeds start at the 1st, 9th, 17th, 25th, 33rd, 41st, 49th, 57th, 65th, 73rd, 81st, 89th, 97th, 105th, 113rd base pair voting to the block, and totals 15 votes. **b** A block having 0 mismatches. In this situation, the first seed of the read voting to the block starts at the 8th base pair, and the 8th, 16th, 24th, 32nd, 40th, 48th, 56th, 64th, 72nd, 80th, 88th, 96th, 104th, 112nd base pair voting to the block, totaling 14 votes. Here, green color stands for a match and red color for a mismatch

Besides, in our algorithm, 31 bp and not 32 bp is selected as seed length is to avoid an unwanted situation where a perfect match block gets less votes than a block with mismatches. In general, we should ideally prefer the perfect match block. We give an example in Fig. 4. Assuming the read length is 150 bp without loss of generality, if the seed length is 32 bp, a block with 2 mismatches, shown in Fig. 4a, could get one more votes than a perfect match block shown in Fig. 4b. The voting strategy selects the block with most votes, while in most situations, the perfect match block shown in Fig. 4b is preferable to the block with mismatches shown in Fig. 4a. Generally, with a read length of 150 and a seed length of 32, the read with the first seed starting at the first seven base pairs or at the eighth base pair will gain at most 15 and 14 votes, respectively.

In this way if the seed length is set as 31 bp, the abnormal voting results caused by 32 bp seed length could be avoided. Each case of exact match will be given 15 votes. If the read length is not 150 bp, FSVA can automatically adjust the seed length to fit the read length by default. Users can manually set seed length also in our tool configuration.

### Mapping quality

For each alignment, FSVA calculates a mapping quality score by comparing the votes of the optimal and the suboptimal blocks. Specifically, the mapping quality score is calculated as following:

$$ mapq= min\left(\left( optimal- suboptimal\right)\times 6,\ 60\right) $$

Where optimal and suboptimal represent the number of votes of the block with the most votes and second most votes, respectively. Obviously our mapping quality score is a multiple of 6 and no more than 60.

## Results

To study the performance of FSVA, we compared FSVA with Subread, BWA and Bowtie2 [15]. BWA is a widely-used read alignment tool based on prefix trie and performs well in practice. BWA has three modes: aln/samse/sampe, bwasw and mem. Here we chose mem, because mem is the best choice for no time cost concern and alignment accuracy for reads with a length more than 100 bp [16]. Bowtie2, a tool from the hash table category, locates a seed using the hash table and implements a single-instruction-multiple-data-accelerated dynamic program to extend the seed. Both BWA and Bowtie2 are very popular in read alignment. Subread is the tool closest to FSVA in its methodology.

In our experiments, when running BWA-MEM, Subread and Bowtie2, all options are set as default. Although FSVA can run on multiple threads, to simplify the comparison of time cost, we ran the test only on single thread for both the simulated data and the real data.

### Evaluation on simulated data

#### Simulated dataset

Our simulated data is produced by wgsim, a tool provided by SAMtools [17]. With the help of wgsim, we can get a set of reads from the reference genome sequence. As the reads are fetched from the reference, we know the exact coordinate of each individual read. Thus, we can compare the predicted location by each alignment tool and the real location to evaluate their accuracy.

Here we use wgsim to fetch 1 million simulated reads from the whole genome sequence hs37d5. Some arguments in Wgsim are set to simulate the properties of reads. To simulate the real situation, we allowed the base error rate be 0.4% and the mutation rate be 0.1%, in which the rate of SNP mutations is 0.085%, and the rate of indel mutation is 0.015%. To study the effect of read length, we generate 125 bp and 150 bp reads respectively in the simulation test.

#### Results on simulated data

As the read is taken from the reference, we know its exact coordinate. If the distance between the real read and the predicted one from a tool is no more than 30 bp, we treat it as a correct alignment.

First, to evaluate the influence of seed length on the final alignment results in FSVA, we did a test on 150 bp reads and 125 bp reads using seeds with different length, and Table 1 shows the result. The comparisons are based on three aspects, time cost, confident mapping percent (with a mapping quality higher than the threshold), and error rate. Obviously, with the seed length increase, the cost of time also increases, and the performance of FSVA is first improved then reduced. To guarantee the speed of FSVA, at the same time taking the situation described by Fig. 4 into consideration, we decided to use 31 bp seed and 30 bp seed respectively when processing 150 bp reads and 125 bp reads, and the test shows FSVA has the best performance using these parameters. Here, 32 bp is the max value of seed length in our program, and users should avoid setting a seed length bigger than 32.

**Table 1:** Evaluation using seeds with different length

rl	125 bp			150 bp		
sl	Time(s)	Conf(%)	Err(%)	Time(s)	Conf(%)	Err(%)
32 bp	146	95.4	0.081	174	96.3	0.048
31 bp				**176**	**96.2**	**0.035**
30 bp	**152**	**95.4**	**0.041**			
23 bp	174	95.5	0.062	213	96.2	0.040
16 bp	218	95.0	0.100	269	96.0	0.072

rl represents read length, sl represents seed length. All the experiments run on a single core of Intel(R) Xeon(R) CPU E5-2670 0 @ 2.60GHz.The bold texts represent t﻿he best performance﻿ on different read length

The overall performances of BWA-MEM, Subread, Bowtie2 and FSVA on simulated data are shown in Table 2. These tests are implemented on 125 bp and 150 bp reads respectively, where the number following the tool name indicates the read length. In regards to time cost, obviously FSVA holds great advantage. The time cost of FSVA on either 125 bp or 150 bp reads and either single-end reads or pair-end reads is much lower than other tools. Concretely, FSVA runs 3–4 times faster than BWA-MEM, 1–2 times faster than Subread and 4–5 times faster than Bowtie2. In time cost considerations, indexing time is not included, because all four tools need to index, which thus is not a main factor for whole genome data processing. Compared with BWA-MEM, FSVA performs a little worse on confident mapping percent and error rate. The difference is not high, 1–2% in confident mapping percent and about 0.02% in error rate. Excluding time cost, on single-end data, performance of FSVA and Bowtie2 are very close, while on pair-end data, the error rate of Bowtie2 is a little higher, 0.297 vs 0.041 and 0.260 vs 0.035. Subread is closest to FSVA in methodology, however the performance of Subread in our test is comprehensively behind FSVA, especially the error rate of Subread, which is too high to be satisfactory.

**Table 2:** Evaluation on simulated data

Tool	sTime(s)	sConf(%)	sErr(%)	pTime(s)	pConf(%)	pErr(%)
BWA-MEM-125	581	96.2	0.030	642	97.8	0.018
Subread-125	227	93.5	0.896	204	95.8	1.268
FSVA-125	170	93.5	0.014	150	95.4	0.041
Bowtie2-125	792	94.9	0.020	772	94.9	0.297
BWA-MEM-150	748	96.7	0.023	737	98.0	0.012
Subread-150	271	95.0	0.728	246	96.9	1.054
FSVA-150	188	95.0	0.014	173	96.2	0.035
Bowtie2-150	966	95.0	0.015	929	94.9	0.260

Except in the ‘Tool’ column, the left three columns starting with ‘s’ represent the performance on single-end data, and the right three columns starting with ‘p’ represent the performance on pair-end data. ‘sTime’, ‘sConf’, ‘sErr’ refer to time cost, confident mapping percent and error rate correspondingly for single-end data. In the first element of each row, such as ‘BWA-MEM-125’, the number 125 following the tool name BWA-MEM represents read length for this test. All the experiments were run on a single core Intel(R) Xeon(R) CPU E5-2670 0 @ 2.60GHz

Consistent with intuitions, longer reads lead to better confident mapping percent, error rate and higher time cost for all tools. For FSVA, the time increase caused by long reads could almost be ignored, which means FSVA will be more competitive with the trend of reads becoming longer and longer.

**Fig. 5:**
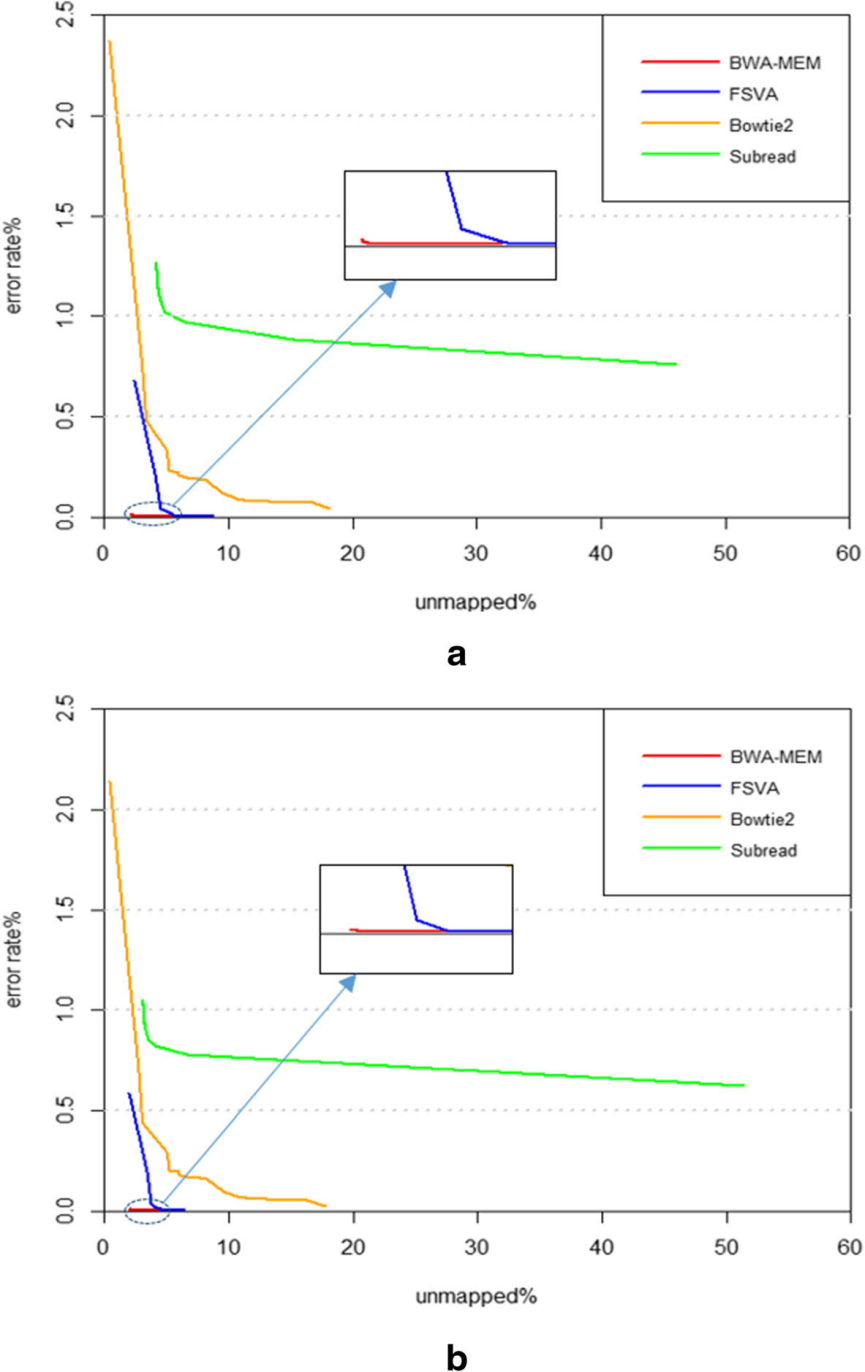
The variety between unmapped percent and error rate on the 125 bp reads (**a**) and the 150 bp reads (**b**). These two figures show the unmapped percent and error rate at each mapping quality level from 1 to 60. The two figures show a similar trend in that the error mapped percentage declines with the rise of unmapped percentage. This is because with the rise of mapping quality threshold, the number of alignments with a mapping quality below the threshold increases, and the alignment with a high mapping quality is less likely to be an error mapping

Figure 5 shows the relationship between unmapped percent and error mapped percent on pair-end data for the 125 bp reads (5(a)) and 150 bp reads (5(b)). Obviously BWA-MEM has the best performance, with both error mapped percent and unmapped percent being very low and varying in a small range. Our FSVA performed a little worse than BWA-MEM and much better than Subread and Bowtie2. When the mapping quality threshold is low, in the range of 1–6, FSVA has a relatively low unmapped percent and a high error mapped percent. With the quality threshold increasing, FSVA’s unmapped percent rises while error mapped percent declines. Noticeably, there is no difference in the error rate between BWA-MEM and FSVA when the mapping quality threshold is higher than 18. This means we can confidently set the threshold as 18 in practice. Under this condition, FSVA has the almost same accuracy as BWA-MEM, except for a slightly lower confident mapping percent. Subread and Bowtie2 show a similar trend as FSVA, but have worse performance than FSVA both on unmapped percent and error mapped percent. Besides, comparing the results on 125 bp vs 150 bp for each tool, we find the performance of all four tools are improved with the increase of read length increases, especially for FSVA. FSVA is more applicable for long reads since longer reads means more seeds, and consequently less uncertainty on voting. Due to this feature, the performance of FSVA is improved further when the read length increases.

Although FSVA is not superior to BWA-MEM in terms of mapping accuracy, its advantage of time saving is extremely significant. In some cases, read data needs to be processed in a short time and the requirement of accuracy is not very stringent, and for this FSVA is undoubtedly the best choice. In next section, tests on real data proves that the difference of accuracy between FSVA and BWA-MEM does not have much influence on downstream variant calling.

As for storage memory, BWA-MEM, FSVA, Subread and Bowtie2 need 5.2, 7.1, 6.7 and 3.2GB respectively, on both 125 bp and 150 bp reads. This level of memory cost can be tolerated by a modern personal computer, let alone a server. Thus, memory cost is not a major concern of read mapping tools.

### Evaluation on real data

#### Real dataset

Five real whole genome sequenced datasets from Illumina HiSeq X10 were used to evaluate these four tools. All the reads were 150 bp and the number of reads varied from ~300 to ~500 million. The library sizes of these five datasets are shown in Table 3.

**Table 3:** Library size of the five datasets

	Dataset1	Dataset2	Dataset3	Dataset4	Dataset5
Library size	201 GB	290 GB	298 GB	284 GB	342 GB

#### Results on real data

Table 4 presents the time cost of BWA-MEM, Subread, FSVA and Bowtie2 on five real data sets shown in Table 3. As in the simulation tests, FSVA is the most time-saving method and this element of FSVA is much more significant (6 ~ 7 times faster) when compared with BWA-MEM. For a genome sequences dataset with a library size of ~300 GB, FSVA requires less than 1 day while BWA-MEM requires almost 6 days. The time cost of FSVA is also much less than that of Bowtie2, almost 6 times less. On the real dataset, Subread cost more time than BWA-MEM and Bowtie2, let alone FSVA. And in the case of big sequencing data (Dataset 2–5 in Table 3), after over 30,000 min (almost 21 days) Subread had only processed one third of the input data. Therefore, it was dropped by us for big data.

**Table 4:** Time cost on real data

Tool	Time1(m)	Time2(m)	Time3(m)	Time4(m)	Time5(m)
BWA-MEM	4443	7783	8311	8056	9181
Subread	8894	-	-	-	-
FSVA	812	1184	1385	1335	1541
Bowtie2	5191	6384	6998	6694	7781

Time cost (minutes) of BWA-MEM, Subread, FSVA and Bowtie2 on real data. These tools all ran on a single core Intel(R) Xeon(R) CPU E5-2670 0 @ 2.60GHz

To study the influence of alignment results on downstream variant calling, we rely on SAMtools. SAMtools is a suite of utilities for interacting with high-throughput sequencing data. One of its utilities is taking output generated by short read aligners like FSVA and BWA-MEM, and calling variants. For each of these four tools, FSVA, BWA-MEM, Bowtie2 and Subread, we consistently utilized SAMtools as variant caller. Then, with some statistical factors of the called variants starting from each aligner, we compared the performance of the four tools. These statistical factors included the number of variants and Ti/Tv ratio. For variants, Ti/Tv is a ratio of the number of transition to transversion substitutions. Recent human studies particularly from the 1000 Genomes Project have been showing that for whole human genome, this ratio should be around 2-2.1. Since Subread did not complete output in the big sequencing datasets (as seen in Table 4), the following analysis is based on Dataset1 of Table 3.

**Fig. 6:**
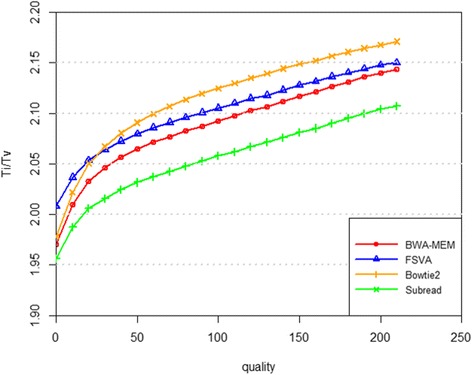
The Ti/Tv ratio of the variants called by SAMtools using the result of BWA-MEM, Subread, Bowtie2 or FSVA. The x-axis stands for the quality of the variant, and the y-axis for the ratio of Ti/Tv

In Fig. 6, relationship between Ti/Tv ratio and called variant quality is shown. In general, higher variant quality means less variants and bigger Ti/Tv ratio. The curves of Ti/Tv to variant quality of FSVA and BWA-MEM are closest, and are in the middle of the curves of Bowtie2 and Subread. In the very low quality region, the Ti/Tv ratio of FSVA is still above 2, better than those of other three tools.

**Fig. 7:**
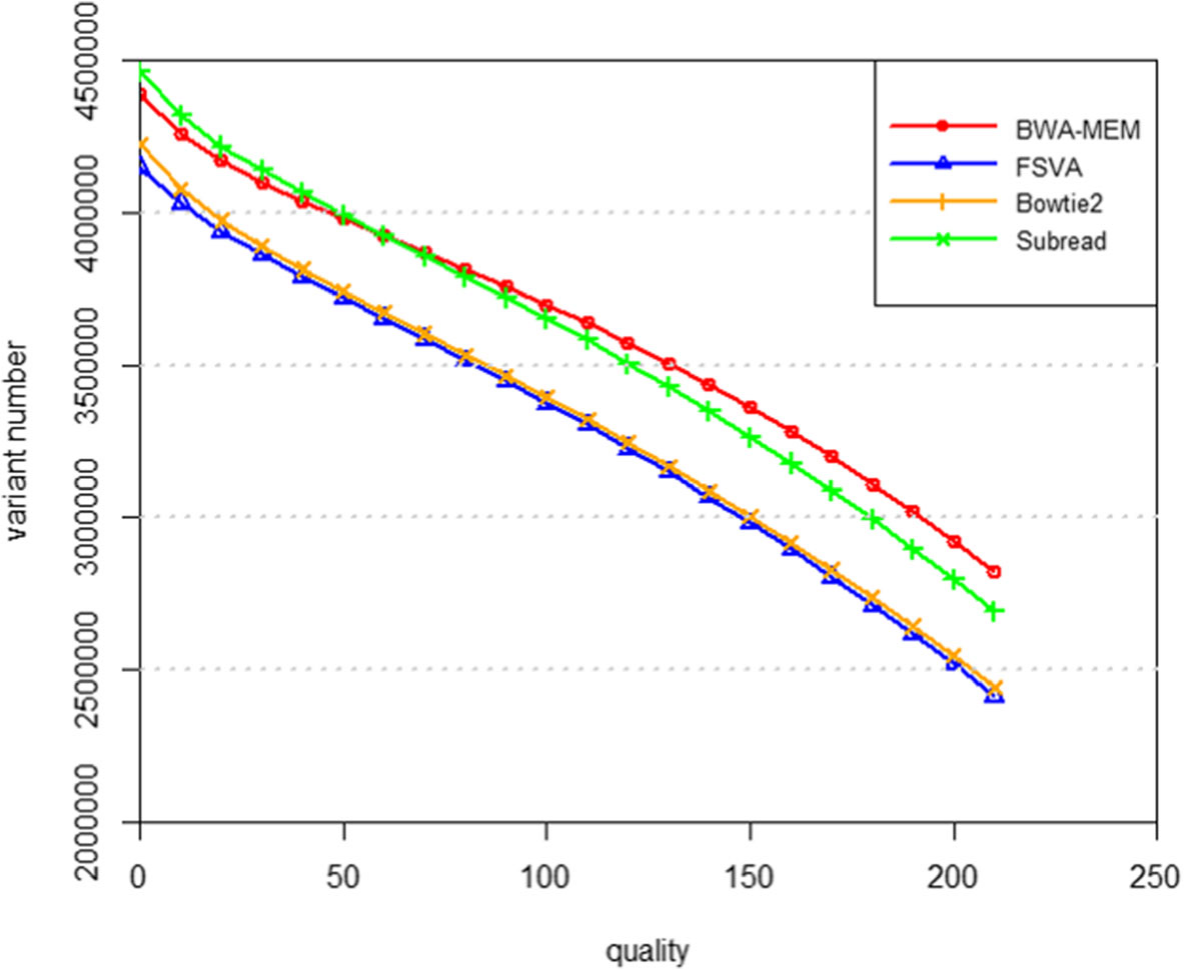
Number of variants called by Samtools using the result of BWA-MEM, Subread, Bowtie2 and FSVA separately. The x-axis stands for the quality of the variant, and the y-axis for the number of variants called by BWA-MEM, Subread, Bowtie2 and FSVA

**Fig. 8:**
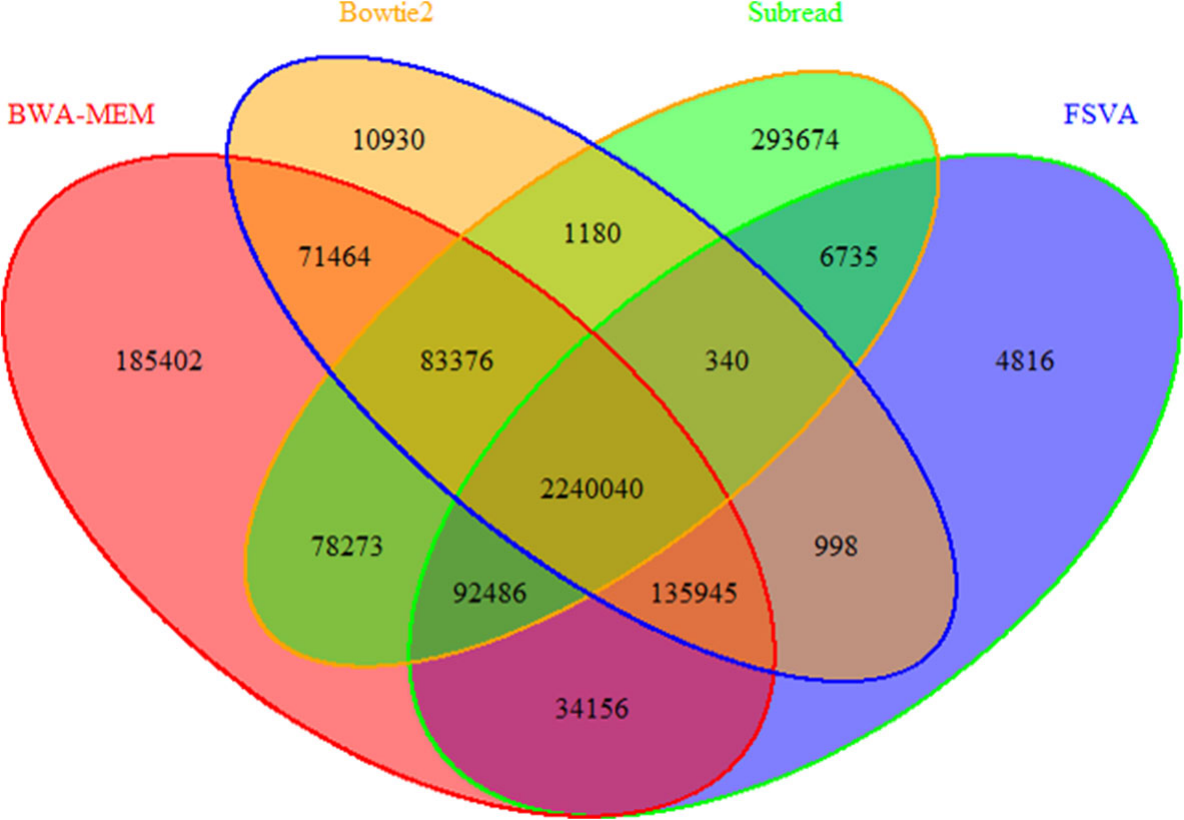
A Venn Diagram of the number of variants with high quality

**Fig. 9:**
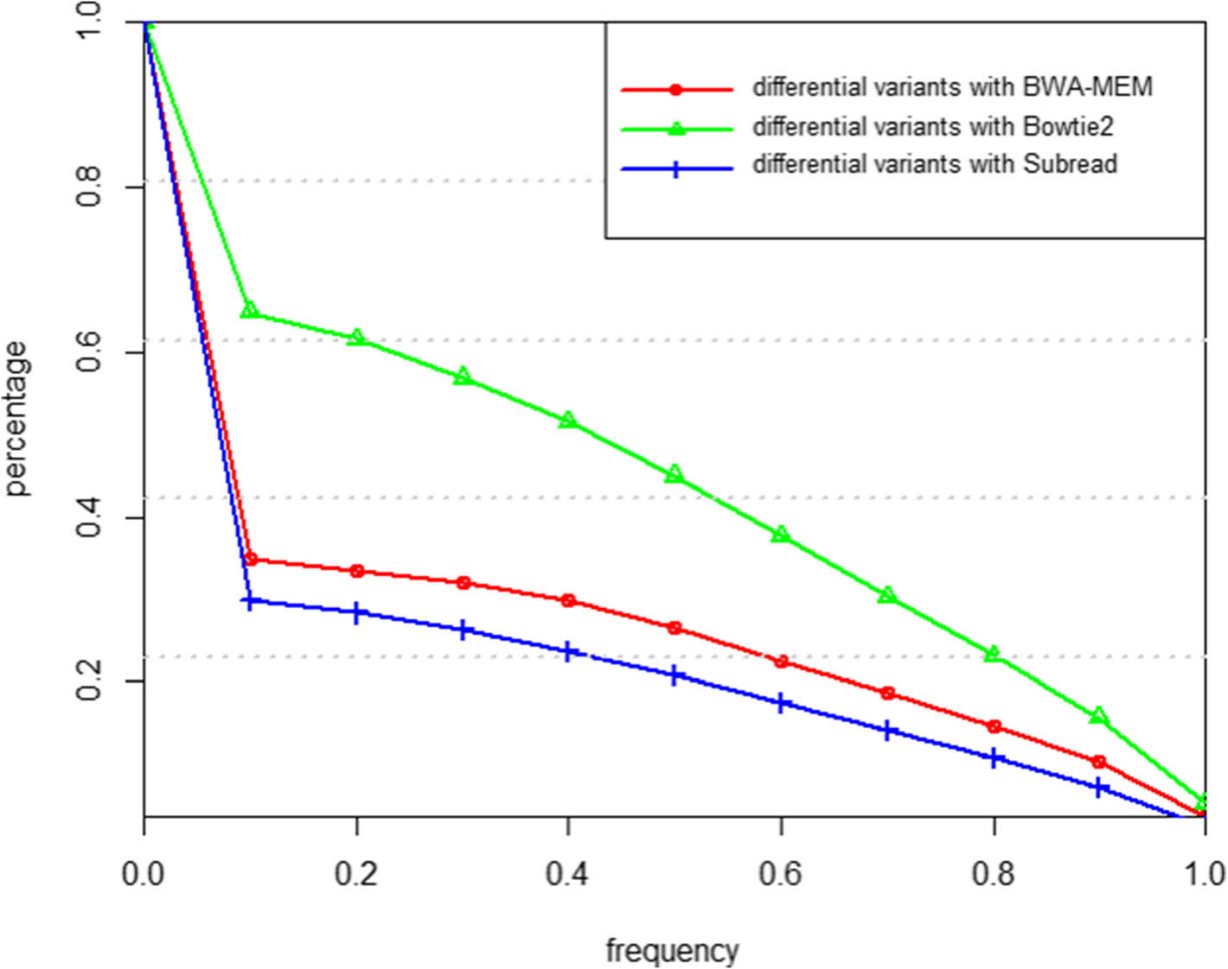
Frequency distribution of the differential variants between FSVA and other tools. The red line, blue line and green line represent variants called via FSVA but not called via BWA-MEM, Subread and Bowtie2, respectively

Figure 7 shows the number of called variants by SAMtools given the alignment results from BWA-MEM, Subread, Bowtie2 and FSVA, respectively. If the variant quality threshold is set as 50, the number of called variants is in the range of 3.6–4 million, and it decreases to around 2 million if the quality threshold is above 200. Overall, for variant calling, BWA-MEM is the tool with most sensitivity, and consequently false positives of BWA-MEM may be more frequent than FSVA and Bowties with high probability. To further illustrate the confidence of called variants of each tool, we present a Venn diagram of the number of variants with high quality (higher than 200) in Fig. 8. For FSVA, the vast majority of the variants can also be called from the alignment output of at least one of the other three alignment tools. Specifically, 89.05% is identified from the results of all the four tools and only 0.19% (4816) cannot be called via any one of other three tools. For BWA-MEM, the corresponding two numbers are 76.68 and 6.35%. For Bowtie2, these numbers are 88.04 and 0.43% correspondingly. The total number of called variants based on Subread is very close to BWA-MEM, while the number of called variants only via Subread is highest, and at 293,674 it is much higher than that of BWA-MEM (185,402). Only 4814 variants are called only via FSVA. This may demonstrate the specificity of called variants based on FSVA is best when compared with the other three tools. The difference between FSVA and each one of the other three tools is studied even further from the point of frequency of called variants in cohort, shown in Fig. 9. Variant frequency is extracted from cohort studies including 1000 Genome Project, ExAC and CHARGE. We can infer that over one third of the variants called via FSVA but not called via BWA-MEM or Subread have a frequency higher than 10%, and almost two third of the variants called via FSVA but not called via Bowtie2 have a frequency higher than 10%.

According to the results of the real data, it is reasonable to say that the read alignment performance of FSVA can be used to get a high-quality variant set. The tests on real data supplement evidence from the simulation test, and show the higher performance of FSVA. Considering both the simulation test and real data test, we believe FSVA is a very competitive read alignment tool.

## Discussion

FSVA utilizes the seed-and-vote strategy. Like most read alignment methods, it builds a hash table for a reference genome first. Then it extracts seeds from each read and searches the hash table to find the location of the seed in the reference genome. The coordinate of a read is voted by the seeds of the read.

The most significant advantage of FSVA is its time saving potential. For a whole set of human genome sequencing data, the time cost of FSVA is one sixth or one seventh of BWA-MEM or Bowtie2. For example, for a sequencing library with 200G size, time cost of FSVA is 13.5 h while BWA-MEM costs 74 h, and Bowtie2 requires 86.5 h on a single CPU core. This impressive feature makes FSVA very competitive in short read alignment with large size, especially for cohort study.

The accuracy of FSVA is illustrated here both on simulation data and real sequencing data. Experiments on simulation data show that the alignment accuracy of FSVA is almost good as BWA-MEM, especially when the mapping quality is selected as over 18, the difference on error rates between BWA-MEM and FSVA is very small, which can basically be ignored. On real sequencing data, since we do not know the correct coordinate of short reads, and usually the main focus of a pipeline for whole genome sequencing data analysis lies on variant calling, read alignment is just the first step. We explored the influence of four mapping tools, FSVA, BWA-MEM, Bowtie2 and Subread, on variant calling. In most cases, variants called based on the results of BWA-MEM are highest, 0.3–0.4 million more than that of FSVA and Bowtie2. For variant calling, BWA-MEM may be the most sensitive, while FSVA appears to have the best specificity. About 99.8% of variants called base on FSVA also could be found based on other short read alignment tools, and 85.67% of variants called based on BWA-MEM could be identified based on FSVA. For a cohort study, where the data involved is almost a tsunami and the accuracy for an individual is not critical, FSVA is a good choice.

FSVA is not suitable for very short reads. As FSVA uses the seed-and-vote strategy, if the read is too short, the extracted seeds should be shortened, otherwise the number of seeds is not enough to vote. But very short seeds are unrepresentative and will introduce too much noise. We suggest FSVA being applied to any library in which read length is over 100 bp. Fortunately, the trend in biotechnology development is towards reads becoming longer and longer.

FSVA is sensitive to SNP and error base. For FSVA, the location of an SNP or an error base will affect the voted output. It is clear enough that an SNP or a base error appearing in the head or tail of a read exists in less seeds than one appearing in the middle of the read. This means an SNP or a base error in the middle of a read will introduce more wrong-voting than if it were at the end of a read. If an indel exists in the middle of a read, the wrong-voting is again worsened. For whole genome sequencing or whole exon sequencing, this problem is alleviated since the reads are randomly cut and an SNP or an indel will be in the middle of some reads and will be at the end of other reads. For amplicon sequencing, since there are a lot of replicate reads, FSVA should be considered more before use.

## Conclusions

In this paper, we proposed a new short read alignment algorithm, named FSVA. FSVA adopts the seed-and-vote strategy, achieving a significant improvement on speed over existing methods. In some cases, reads have to be aligned in a short time and requirements of accuracy are not very stringent. In these incidences, FSVA would be a good choice.

## Abbreviations

BWA: Burrows-Wheeler Alignment tool
FSVA: Fast Seed-and-Vote Aligner
